# The Impact of Acute Mild Normobaric Hypoxia and a Single Bout of Exercise to Volitional Exhaustion on Cognitive Performance in Endurance and Strength-Trained Athletes: The role of BDNF, EP-1, Catecholamines and Lactate

**DOI:** 10.5114/jhk/168282

**Published:** 2023-07-15

**Authors:** Zofia Piotrowicz, Miłosz Czuba, Małgorzata Chalimoniuk, Józef Langfort

**Affiliations:** 1 Institute of Sport Sciences, The Jerzy Kukuczka Academy of Physical Education, Katowice, Poland.; 2 Department of Applied and Clinical Physiology, Collegium Medicum University of Zielona Gora, Zielona Gora, Poland.; 3 Department of Physical Education and Health in Biała Podlaska, Józef Piłsudski University of Physical Education in Warsaw, Biała Podlaska, Poland.

**Keywords:** psychomotor performance, brain-derived neurotrophic factor, exhaustion, athletes

## Abstract

The aim of the study was to examine whether a single bout of exercise to volitional exhaustion, performed under moderate normobaric hypoxia (H), would affect psychomotor performance (PP) in differently trained athletes. For this purpose, ten strength-trained (S) athletes, ten endurance-trained (E) athletes and ten healthy men leading a sedentary lifestyle as a control (C) group performed voluntarily two graded exercise tests until volitional exhaustion (EVE) under normoxia (N) and H (FiO_2_ = 14.7%). We measured the peripheral level of the brain derived neurotrophic factor (BDNF), choice reaction time (CRT) and the number of correct reactions (NCR) as indices of PP. Psychomotor tests were performed at rest, immediately after the EVE and 3 minutes after the EVE. Venous blood samples were collected at rest, immediately after cessation of each EVE, and 1 h after each EVE. The results showed that the EVE significantly (p < 0.05) impaired CRT under N and H, and NCR under H only in the E group. The higher WR_max_ in the E compared to the S and C groups was associated with a significant (p < 0.005) increase in adrenaline (A) and noradrenaline (NA). There were no significant differences between conditions (N vs. H) in the BDNF at rest and after exercise. The EVE impaired cognitive function only in the E group; higher involvement of the sympathetic nervous system, A and NA may also play a role in this phenomenon. Therefore, it can be concluded that exposure to H did not have a negative impact on CRT or NCR. Moreover, BDNF did not improve cognitive function.

## Introduction

Exercise and exposure of the human body to hypoxia are well-known physiological and environmental stressors that can modify the functions of the central nervous system (CNS), but typically their effect is the opposite. Hypoxia is thought to have detrimental effects on the CNS by decreasing arterial pressure of O_2_ (PaO_2_) and arterial saturation of O_2_ (SaO_2_) (Kolb et al., 2004) leading to neurological deficits and impairment of cognitive performance at rest (Wilson et al., 2009). Furthermore, the turnover of several neurotransmitters can be altered under hypoxia compared to normoxia, adversely affecting cognitive functions (Gibson et al., 1981; Kramer et al., 2006; Levitt and Gutin, 1971). Less clear-cut results relate to physical effort under hypoxia, as under such conditions both beneficial and detrimental effects on cognitive performance have been reported. This applies primarily to research published in recent years: some studies have reported cognitive improvement in response to combined effects of acute exercise and hypoxia (Komiyama et al., 2017; Seo et al., 2017), whereas other studies have found that cognitive performance was impaired under such conditions (Lefferts et al., 2016; Ochi et al., 2018). Nevertheless, there does seem to be a research-based consensus, supported by a growing body of evidence, suggesting that acute exercise at moderate intensity under hypoxia does indeed improve cognitive performance (Chang et al., 2012). The aforementioned phenomenon may be, at least partially, explained by increased cardiac output and cerebral blood flow, which are pivotal in maintaining oxygen delivery to the CNS (Aimslie and Subudhi, 2014). The above described inconsistent findings were above all obtained in studies conducted in young sedentary men, and the resulting differences are attributed to methodological differences and differing experimental designs (Dinoff et al., 2016).

Brain-derived neurotrophic factor (BDNF), which is believed to affect cognitive functions, among other things, is the most abundant neurotrophin located in the hippocampus, cortex, midbrain, thalamus (amygdala), hypothalamus, striatum, pons and medulla (Binder and Scharfman, 2004; Cunha et al., 2010). Importantly, dopaminergic neurons of the substantia nigra and striatum, e.g., structures involved in movement regulation, have been found to be the main source of BDNF secretion (Ventriglia et al., 2013). Regular long-term repeated physical exercise and/or moderate to high intensity training induces an increased level of BDNF and TrkB receptors in the brain regions responsible for motor activity (Zoladz and Pilc, 2010). Acute aerobic exercise increases the concentration of the BDNF in the blood (Szuhany et al., 2015) in both trained (Piotrowicz et al., 2020) and sedentary individuals (Piotrowicz et al., 2019). This increase depends on the intensity and duration of the single bout of exercise or the training process (Liu and Nusslock, 2018). In other studies, an increase in serum BDNF was reported after high-intensity interval training (HIIT) (Helm et al., 2017). Notably, high-intensity exercise performed at short intervals has been found to be more effective than continuous high-intensity exercise for increasing serum BDNF (Jiménez-Maldonado et al., 2018), while a 12-week resistance training exercise program of healthy individuals was not found to cause an increase in basal BDNF concentrations (Williams and Ferris, 2012). Keeping in mind that the vast majority of studies have provided evidence of the beneficial effects of physical exercise on cognitive functions, with a simultaneous increase in BDNF levels in the blood, this suggests that BDNF may be involved in improving cognitive function (Ferris et al., 2007; Gold et al., 2003; Hung at al., 2018).

One of the first studies on BDNF release during exercise under hypoxia showed no additive effect on the serum BDNF concentration (Van Cutsem et al., 2015). A previous study (Piotrowicz et al., 2020) reported that maximal exercise performed under both normoxia and hypoxia conditions, corresponding to 3000 m altitude (FiO_2_ = 14.7%), caused a similar increase in peripheral BDNF concentration in endurance athletes. However, an increase in the BDNF did not prevent cognitive impairment under hypoxia, as measured by choice reaction time (CRT) and the number of correct reactions (NCR) (Piotrowicz et al., 2020). These limited data indicate that the relationship between BDNF secretion and cognitive function under hypoxia is unclear and poorly understood.

The effects of habitual exercise or training processes on the human body are thought to be the result of repeated exercise, and therefore may be associated with cumulative acute responses to exercise. Importantly, the chronic effects of exercise can be modified using the same acute exercise, by changing the exercise mode, strength, duration, and/or frequency. One can expect that this may also involve different adaptive changes in the brain in response to different training processes. Therefore, adaptive changes in the brain triggered by strength training may be different from those caused by endurance training (Hashimoto et al., 2021). For example, studies demonstrated that some metabolic factors (e.g., lactate, myokines), released during muscle contraction depending on the exercise mode, activated unmyelinated group IV afferents, whereas mechanical-sensitive, myelinated group III afferents were preferentially stimulated during the production of muscle strength (Gandevia, 2001). Studies in animals have revealed that adaptive changes in the brain triggered by endurance training affect the serotonergic system (Langfort et al., 2006). Other research on the effects of aerobic and strength training on BDNF levels and neuroplasticity has found that both endurance and resistance-training result in similar stimulating effects on BDNF levels in rats (Vilela et al., 2017).

It is not known whether acute exercise to exhaustion under hypoxic conditions will elicit a different response in individuals who exhibit adaptive changes in the brain caused by endurance or resistance training. This phenomenon is not yet fully understood considering psychomotor skills and possible involvement of BDNF in this phenomenon due to a small number of studies. In this regard, the aim of the present study was to examine whether a single bout of maximal exercise to volitional exhaustion, performed under moderate normobaric hypoxia, would affect psychomotor performance in strength-trained athletes, compared to endurance-trained athletes and individuals leading a sedentary lifestyle. For this purpose, we measured the peripheral level of BDNF, CRT and NCR as indices of psychomotor performance during exercise to volitional exhaustion. In addition, we examined the level of selected circulating biochemical factors, such as cortisol (COR), lactate (LA), nitric oxide (NO), endothelin-1 (ET-1), and catecholamines, because they are known to affect BDNF expression / production, and their expression can be influenced by both exercise and hypoxia.

## Methods

### Participants

Ten strength-trained (S) athletes (age 22.0 ± 1.7 years; body height 180.7 ± 3.9 cm; body mass 79.6 ± 6.4 kg; fat content 11.3 ± 4.0%) and ten endurance-trained (E) athletes (age 21 ± 1.3 years; body height 180.2 ± 6.7 cm; body mass 69.6 ± 6.8 kg; fat content 9.1 ± 3.1% ), as the experimental groups, plus ten healthy men leading a sedentary lifestyle (age 20.1 ± 1.2 years; body height 177.2 ± 8.9 cm; body mass 71.4 ± 10.6 kg; fat content 12.1 ± 4.5%), as a control (C) group, were recruited for the study. All participants had current valid medical examinations and showed no contraindications to participating in the experiment.

Each participant declared that for at least one month prior to testing, they had not taken either medications or dietary supplements, and each provided written informed consent prior to the study commencement. The experimental procedures and related risks were explained to all the participants verbally, and they were able to withdraw from the study at any time. The investigation was conducted according to the Helsinki Declaration and was approved by the Ethics Committee for Scientific Research at the Jerzy Kukuczka Academy of Physical Education in Katowice, Katowice, Poland (no. 5/2013, approval date: 26 June 2013).

Participants were tested on two occasions, five days apart. On each occasion, participants underwent two graded exercise tests until volitional exhaustion (EVE) on an ergocycle, performed under normobaric normoxic and normobaric hypoxic conditions (3000 m asl). Participants were allocated to the normoxic or hypoxic conditions randomly, by means of a computer-generated list (Urbaniak and Plous, 2013). Before each test, the body mass and body composition of each participant was determined using electrical impedance measurements, by means of an Inbody 720 body composition analyzer (Biospace Co., Tokyo, Japan).

To create hypoxic conditions, representing the equivalent of 3000 m asl altitude (FiO_2_ = 14.7%), we used a normobaric hypoxia chamber (Losa Hyp/Hyop-2/3NU system, Lowoxygen Systems, Berlin, Germany) maintained at the Hypoxia Laboratory.

### Ergocycle Graded Exercise Test to Volitional Exhaustion

The graded exercise tests were carried out on an Excalibur Sport ergocycle (Lode BV, Groningen Netherlands), beginning at a workload of 40 W, which was increased by 40 W every 3 min until volitional exhaustion. When a particular participant discontinued the test before completing a given workload, then the maximum workload for that test was calculated by the formula WR_max_ = WR_k_ + (t/T × WR_p_) (Kuipers et al., 1985), where *WR_k_* represented the previous workload, *t* represented exercise duration with the workload until premature failure, *T* represented duration of each workload, and *WR_p_* was the workload increment by which exercise intensity was increased during the test.

During the tests, the heart rate (HR), minute ventilation (VE), breathing frequency (BF), oxygen uptake (VO_2_) and carbon dioxide content in expired air (VCO_2_) were recorded for each participant by means of a MetaMax 3B gas analyzer (Cortex, Leipzig, Germany). Fingertip capillary blood samples for the assessment of LA concentration (Biosen C-line Clinic, EKF- diagnostic GmbH, Barleben, Germany) were drawn at rest and at the end of each testing stage.

### Psychomotor Performance Determination

Choice reaction time and NCR were selected as indices of psychomotor performance. The CRT console was mounted on the wall in front of the ergometer, at the eye level, 1.5 m away from the participant. The test included 15 positive (red light or a sound) and 15 negative (green or yellow light) stimuli displayed in randomized order, in 1s to 4 s intervals. Participants were asked to press and then to release, as quickly as possible, the button of the switch device kept in their right hand in response to the red light stimulus, the button in the left hand in response to the sound stimulus, and not to react in either way to the negative stimuli. The total time for each CRT was 107 s. The stimuli and participants’ responses were recorded using the reaction time measuring device (MRK 432, ZEAM, Zabrze, Poland), and determined to the nearest 0.01 s. The results are presented as the mean reaction time for all 15 responses to positive stimuli. Participants had been familiarized with the testing procedure a week before the study, by practicing the task both at rest and during cycling.

### Venous Blood

On the day of the EVE, each participant was cannulated into their antecubital vein, 15 min prior to having breakfast. Two samples of venous blood were then collected at each time point: 10 min later, immediately after cessation of each ergocycle test, and again 1 h after each ergocycle test. Of each pair of samples, one was drawn using an ethylenediaminetetraacetic (EDTA) tube (for morphology analysis); the other was drawn using an anti-coagulant tube and processed for serum for the other biochemical assays (BDNF, ET-1, C, catecholamines). After 30 min, blood samples were centrifuged at 1500 × g for 15 min. The serum samples so obtained were kept stored at −80°C until they were analyzed.

### Determination of Brain-Derived Neurotrophic Factor, Cortisol and Endothelin-1 Concentrations

Assays of blood serum levels of BDNF, C, and ET-1 were performed using a commercially available Quantikine ELISA kit (R&D Systems, Minneapolis, MN, USA) in compliance with the procedure supplied by the manufacturer. This method allows for measurements of BDNF, C, and ET-1 in the range of 0.372–4000 pg/mL, 0.030–100 ng/mL, and 0.031–50 ng/mL, respectively. The intra-assay coefficient of variance was <4.0%, <8%, <4%, respectively. To quantify the level, a standard curve was performed using a standard solution.

### Determination of Catecholamines by HPLC Method

Assays of blood serum levels of adrenalin (A), noradrenaline (NA) and dopamine (DA) were carried out using a high-performance liquid chromatograph (HPLC, Gynkotek, Copenhagen, Denmark) with electrochemical detection using a Coulochem III model 520 (ESSA, Copenhagen, Denmark). Serum samples were mixed with 0.1 M perchloric acid containing 22.5 ng/mL ascorbic acid (ASC, Sigma-Aldrich, St. Louis, MO, USA). After being centrifuged at 15,000 g for 10 min at 4°C, the supernatant was filtered through a nylon syringe filter (Millipore, 0.22 μm, Merck KGaA, Darmstadt, Germany). Samples of 20 μL filtrate were then injected into a high-performance liquid chromatography system (Gynkotek, Copenhagen, Denmark) equipped with a Hypersil Gold (15 cm × 4.6 mm) column (Thermo Electron Corporation, Kleinostheim, Germany). The samples were eluted by a mobile phase comprising 107 mM of Na_2_HPO_4_ × 2H_2_O, 107 mM citric acid, 0.3 mM octane-1 sulfonic acid sodium salt (OSA), 0.2 μM of EDTA, pH, 4.6, 1.5% methanol and 1.5% acetonitrile, at a flow rate of 0.8 mL/min. The column temperature was set at 25°C. Peaks were identified by electrochemical detection (Coulochem III, ESSA, Copenhagen, Denmark) with potentials set at E1 = −50 mV and E2 = +400 mV. Data were collected and analyzed using Chromeleon software running on a PC (Gynkotek, Copenhagen, Denmark). DA and 5-HT levels in the sample were calculated by extrapolating the peak area from a standard curve.

### Determination of Nitric Oxide in Serum

Nitrate/nitrite (NO) concentration was determined in serum using a Total Nitric Oxide and Nitrate/Nitrite assay (kat. No.KGE001; R&D Systems, Minneapolis, MN, USA) according to the manufacturer’s instruction. This assay determines nitric oxide concentrations based on the enzymatic conversion of nitrate to nitrite by nitrate reductase. The reaction is followed by colorimetric detection of nitrite as an azo dye product of the Griess Reaction. The Griess Reaction is based on the twostep diazotization reaction in which acidified NO_2_^-^ produces a nitrosating agent, which reacts with sulfanilic acid to produce the diazonium ion. This ion is then coupled to N-(1-naphthyl) ethylenediamine to form the chromophoric azo-derivative which absorbs light at 540–570 nm. The optical density (OD) in each 96-well plate was measured using a microplate reader (Ex 800 TK Biotech, Taipei City, Taiwan) at 540 nm. The concentration of nitrite was calculated by the nitrite standard curve.

### Statistical Analysis

The results of the study were analyzed using Statistica 13.3 software (TIBCO Software Inc., USA). The results are presented as arithmetic means (x) and standard deviations (SD). The statistical significance was set at *p* < 0.05. Prior to all statistical analyses, the normality of the distribution of variables was verified using the Shapiro-Wilk test. The intergroup differences between the research trials, comparisons of repeated measurements and differences between the groups were assessed by the analysis of variance (ANOVA) for repeated measures. When significant differences were found, the Tukey’s post-hoc test was used.

## Results

### Incremental Test Results and Blood Lactate Concentration

ANOVA with repeated measures showed a significant interaction (conditions × groups) for the maximal workload (WR_max,_ F = 3.5; *p* < 0.05) and VO_2max_ (F = 91.2; *p* < 0.001) during the incremental test. Statistical analysis did not show a significant interaction (conditions × groups) for delta values of blood lactate concentration (∆LA) during the incremental test; however, ∆ values of LA in hypoxia were significantly different compared to normoxia (F = 40.09, *p* < 0.001).

**Figure 1:**
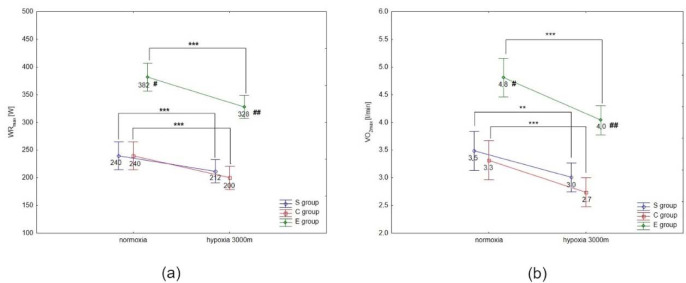
Mean values of the maximal workload (WR_max_) (a) and VO_2max_ (b) during the incremental test performed under different conditions. S – strength-trained; E – endurance-trained; C – control; *** p < 0.001 (compared to normoxia); ** p < 0.001 (compared to normoxia); # p < 0.01 (compared to C and S groups under normoxia), ## p < 0.01 (compared to S and C groups under hypoxia)

**Figure 2:**
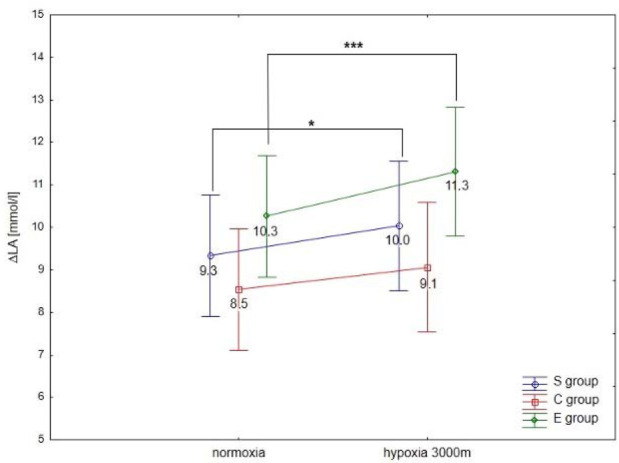
Mean values of ∆LA after the incremental test performed under different conditions. * p < 0.05 (compared to normoxia); *** p < 0.001 (compared to normoxia)

The Tukey’s post-hoc test revealed that WR_max_ (*p* < 0.001) decreased significantly due to hypoxia exposition (3000 m) in all groups (by 14.1% in E athletes, 11.7% in S athletes, and 16.7% in the C group) (Figure 1a). Moreover, WR_max_ values in the E group were significantly (*p* < 0.01) higher compared to the S and C groups under normoxia and hypoxia (Figure 1a). The same trend of changes was also observed in VO_2max_ values under hypoxia. The statistical analysis showed a significant (*p* < 0.001) decrease in VO_2max_ values under hypoxia (3000 m) in all tested groups (by 16.7% in E athletes, 14.3% in S athletes, and 18.2% in the C group; Figure 1b). The values of VO_2max_ in the E group were significantly (*p* < 0.001) higher compared to the values in S and C groups under normoxia and hypoxia (Figure 1b). However, ∆LA during the incremental test under hypoxia significantly (*p* < 0.05) increased in S and E groups, by 7.5% and 9.7%, respectively, compared to normoxia (Figure 2). There were no statistically significant changes in ∆LA values in the C group under hypoxia.

### Choice Reaction Time and the Number of Correct Reactions

ANOVA with repeated measures for time × group interactions showed statistically significant differences in CRT under normoxia (F = 15.46, *p* < 0.001) and hypoxia. Similarly, there was a significant difference in NCR under normoxia (F = 3.98; *p* < 0.0) and hypoxia (3000 m) (F = 18.18; *p* < 0.001).

**Figure 3:**
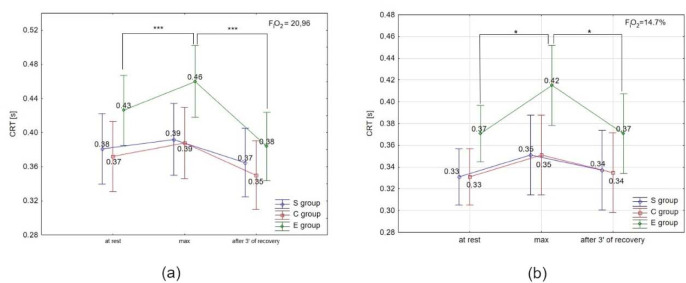
Choice reaction time (CRT) at rest, during maximal effort (max) and after 3 min of recovery under (a) normoxia, and (b) hypoxia (3000 m). *** p < 0.001

The Tukey’s post-hoc test revealed a significant (*p* < 0.05) increase in CRT immediately after the test, and a significant (*p* < 0.05) decrease after 3 min of recovery only in the E group under normoxia and hypoxia (Figures 3a, 3b). There were no significant differences in CRT in the S and C groups under either condition, as well as between conditions (normoxia vs. hypoxia) in all groups.

**Figure 4:**
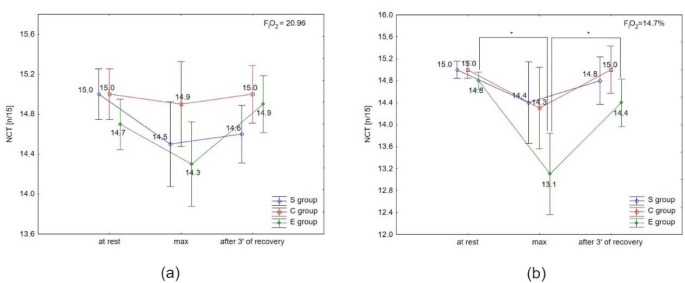
Number of correct reactions (NCR) at rest, during maximal effort (max) and after 3 min of recovery in (a) normoxia, and (b) hypoxia (3000 m). * p < 0.05

There were no significant differences in NCR in the S and C groups under either condition, or between conditions (normoxia vs. hypoxia) in all groups (Figures 4a and 4b). The post-hoc test showed a significant (*p* < 0.05) decrease in NCR immediately after the test, and a significant (*p* < 0.05) increase after 3 min of recovery only in the E group under hypoxia (Figure 4b).

### Brain Derived Neurotrophic Factor and Selected Biochemical Variables

ANOVA with repeated measures for time × group interactions showed statistically significant differences in BDNF concentration under normoxia (F = 2.55, *p* < 0.05) and hypoxia (F = 34.93, *p* < 0.001), ET-1 concentration under normoxia (F = 4.27; *p* < 0.01) and hypoxia (F = 3.53; *p* < 0.05), NO_2_^-^ under normoxia (F = 5.01; *p* < 0.01) and hypoxia (F = 10.72; *p* < 0.0001), COR only under normoxia (F = 2.89; *p* < 0.06), and A under normoxia (F = 8.18; *p* < 0.001) and hypoxia (F = 18.75; *p*< 0.001). There were no significant time × group interactions for DA and NA concentration under normoxia and hypoxia. However, NA concentration under hypoxia was significantly different (F = 12.62, *p* < 0.001) compared to normoxia.

ANOVA with repeated measures did not show a significant condition × group interaction for BDNF concentration at rest, immediately after the test and after 1 h of recovery; however, BDNF concentration immediately after the test under hypoxia was significantly different (F = 11.48, *p* < 0.01) compared to normoxia.

**Figure 5:**
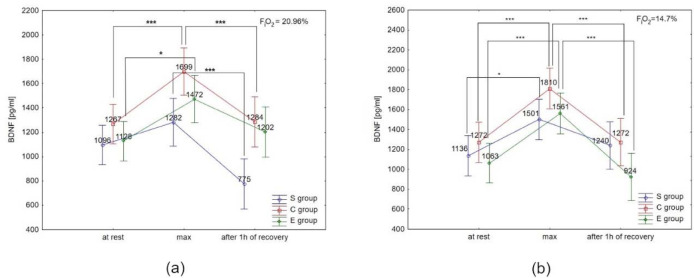
Brain-derived neurotrophic factor (BDNF) serum concentration at rest, immediately after the test (max) and after 1 h of recovery under (a) normoxia, and (b) hypoxia (3000 m). *p < 0.05; *** *p* < 0.001.

The Tukey’s post-hoc test showed that BDNF concentration under normoxia significantly increased immediately after the test in the E group (by 30.5%; *p* < 0.05) and the C group (by 34.1%; *p* < 0.01). Additionally, BDNF concentration decreased significantly (*p* < 0.01) after 1 h of recovery, by 24.4% in the C group, and 39.5% in the S group under normoxia (Figure 5a). Under hypoxia conditions (3000 m), BDNF concentration increased significantly (*p* < 0.05) immediately after the test in all groups (by 46.8% in E athletes, 32.1% in S athletes, and 42.3% in the C group). After 1 h of recovery, BDNF concentration significantly decreased (*p* < 0.001) by 40.8% in the E group, and by 29.7% in the C group. There was no significant decrease in BDNF concentration after 1 h of recovery in the S group in the hypoxia environment (Figure 5b). The Tukey’s post-hoc test revealed that BDNF concentration immediately after the test in the E group was significantly higher (by 6.5%, *p* < 0.05) under hypoxia compared to normoxia.

Additionally, the post-hoc test showed that ET-1 significantly increased immediately after the test (by 44.9%, *p* < 0.001), and significantly decreased after 1 h of recovery (by 47.8%, *p* < 0.001), compared to the resting level in the C group under normoxia (Table 1). Under hypoxia, ET-1 increased significantly immediately after the test only in the C group (by 35.2%, *p* < 0.001). There were no significant differences in other groups in ET-1 concentration (Table 2).

**Table 1:** Mean values of selected biochemical variables registered under normoxia condition at rest, immediately after the incremental test (max) and after 1 h of recovery (after 1 h).

Variables		Normoxia					
		at rest		max		after 1 h	
	group	x ± SD	Me	x ± SD	Me	x ± SD	Me
**ET-1** [pg/ml]	S	6.8 ± 4.5	4.8	8.4 ± 4.1	6.5	6.7 ± 4.0	5.4
	E	2.5 ± 0.9	2.5	2.4 ± 1.8	2.3	2.0 ± 1.1	1.8
	C	6.9 ± 3.1	6.7	10 ± 2.8***	11.3	10.2 ± 4.7**	8.6
**NO_2_^-^** **[pg/ml]**	S	41.5 ± 28.5	28.7	39.7 ± 24.3	28.1	47.1 ± 13.5	46.8
	E	29.8 ± 12.2	27.5	47.7 ± 22.8	36.9	31.5 ± 12.6	25.7
	C	49.1 ± 21.5	54.9	59.5 ± 20.6	48.2	**31.7 ± 18.2#**	27.7
**C** **[pg/ml]**	S	7.1 ± 2.7	6.3	11 ± 3.4***	11.1	**7.9 ± 3.1#**	7.4
	E	7.8 ± 3.0	6.7	11.1 ± 3.5	11.3	9.4 ± 2.9	9.7
	C	7.8 ± 3.0	6.6	11.0 ± 3.5	10.3	9.5 ± 2.9	9.7
**NA** **[pg/l]**	S	127.9 ± 72.0	136.3	263.1 ± 126.8	240.7	262.5 ± 93.4	237.1
	E	363.6 ± 207.8	366.4	**566.8** ± 261.3**	575.6	422.1 ± 212.9	471.8
	C	364.5 ± 133.3	350.3	439.9 ± 123.8	422.1	442.3 ± 89.7	402.6
**A** [pg/l]	S	18.6 ± 12.6	14.1	189.5 ± 92.7**	165.6	174.3 ± 50.1***	163.1
	E	53.9 ± 31.0	46.0	383.5 ± 197.2***	351.8	**122.5 ± 102.0##**	117.0
	C	31.9 ± 8.0	29.2	188.5 ± 32.0***	189.5	133.4 ± 22.1	139.7
**DA** [pg/l]	S	12.0 ± 7.2	11.8	17.5 ± 10.1	13.5	16.3 ± 10.8	13.1
	E	4.3 ± 1.7	4.2	8.7 ± 6.1	7.6	5.4 ± 1.3	5.2
	C	13.8 ± 2.5	14.3	19.9 ± 4.2	18.8	14.8 ± 1.4	15.1

S – strength-trained; E – endurance-trained; C – control; * p < 0.05; ** p < 0.01; *** p < 0.001 compared to rest; # p < 0.01; ## p < 0.001 compared to max; & p < 0.08 compared to rest

**Table 2:** Mean values of selected biochemical variables registered under hypoxia condition at rest, immediately after the incremental test (max) and after 1 h of recovery (after 1 h).

Variables		Hypoxia 3000 m					
	group	at rest		max		after 1 h	
		x ± SD	Me	x ± SD	Me	x ± SD	Me
**ET-1** [pg/ml]	S	6.8 ± 3.3	6.4	8.1 ± 2.7	7.5	8.2 ± 3.4	7.4
	E	2.3 ± 0.8	2.5	2.3 ± 1.0	2.5	2.6 ± 1.2	2.3
	C	9.1 ± 4.0	7.6	12.3 ± 5.8***	9.7	10.6 ± 3.9	11.9
**NO_2_^-^** **[pg/ml]**	S	46.7 ± 21.7	36.2	38.9 ± 6.6	42.6	51.8 ± 23.1	41.8
	E	35.0 ± 16.7	40.1	53.3 ± 16.6*	51.0	**30.2 ± 9.5#**	28.0
	C	29.2 ± 12.3	31.0	57.3 ± 23.8***	56.6	**22.4 ± 5.5#**	23.0
**C** **[pg/ml]**	S	6.1 ± 2.4	6.1	9.1 ± 2.6	8.9	8.1 ± 9.2	5.9
	E	6.6 ± 2.3	6.4	8.3 ± 2.5	8.8	8.1 ± 4.3	7.1
	C	6.6 ± 2.2	6.4	9.9 ± 2.0	9.2	9.4 ± 2.9	9.7
**NA** **[pg/l]**	S	367.0 ± 198.9	470.9	732.3 ± 326.9	746.6	639.9 ± 231.5	588.4
	E	326.3 ± 158.9	238.6	1047.6 ± 1080.6*	348.4	591.5 ± 374.1	471.8
	C	149.9 ± 43.2	136.0	392.3 ± 167.7	336.6	416.9 ± 78.4	399.6
**A** [pg/l]	S	31.5 ± 13.5	25.5	327.6 ± 91.5**&**	318.9	301.1 ± 130.0	333.6
	E	204.4 ± 74.5	228.5	1498.1 ± 627.0**	1539.1	856.9 ± 261.4*#	763.7
	C	36.2 ± 7.4	37.9	271.3 ± 67.0	302.5	191.4 ± 40.7	198.9
**DA** [pg/l]	S	42.8 ± 20.0	38.3	50.7 ± 25.8	39.1	56.8 ± 21.5	55.8
	E	7.3 ± 2.6	6.8	12.5 ± 2.9	14.3	12.6 ± 5.5	13.7
	C	13.5 ± 3.7	15.1	20.1 ± 2.5	21.5	16.7 ± 1.3	16.9

S – strength-trained; E – endurance-trained; C – control; * p < 0.05; ** p < 0.01; *** p < 0.001 compared to rest; # p < 0.01; ## p < 0.001 compared to max; & p < 0.08 compared to rest

Under normoxia, NO_2_^-^ decreased significantly (*p* < 0.01) by 46.7%, after 1 h of recovery compared to values after exercise in the C group (Table 1). Hoverer, under hypoxia, NO_2_- increased significantly (*p* < 0.05) immediately after the test in the E and C groups, by 52.3% and 96.2%, respectively. After 1 h of recovery under hypoxia condition, NO_2_^-^ concentration decreased significantly (by 43.3%, *p* < 0.01) only in the E group. Serum concentration of COR increased significantly (by 56.3%, *p* < 0.001) immediately after the test compared to initial concentration in the C group, and decreased significantly (by 28.8%, *p* < 0.001) in this group after 1 h of recovery(Table 2). There were no significant differences under normoxia and hypoxia in other examined groups. Concentration of NA increased significantly (*p* < 0.01) immediately after the test by 55.9% in the E group under normoxia, and by 221.1% under hypoxia. There were no significant differences in other groups under all conditions (Tables 1 and 2). Under normoxia, levels of A increased significantly (*p* < 0.001) immediately after the test in all groups (611.5% in E athletes, 918.8% in S athletes, 491% in the C group). Moreover, in the S group, after 1 h of recovery, A concentration was significantly (*p* < 0.001) lower than at rest, by 837.1%, and in the E group by 68.1% compared to the level after exercise. Under hypoxia, statistical differences in the A level were only found in the E group. After exercise, the A level increased significantly (*p* < 0.001), by 632.9%, and decreased significantly (*p* < 0.001) after 1 h of recovery, by 42.8%. Additionally, in the S group, there was a tendency (*p* < 0.08) towards an increase in the A level immediately after the test under hypoxia, by 940%.

## Discussion

Among the factors that have an influence on the combined effects of exercise and hypoxia on cognitive performance, a pivotal role is attributed to the severity or/and duration of hypoxia, as well as to the intensity or/and duration of exercise (Taylor et al., 2016). Less recognized and poorly investigated is the influence of the type of exercise and the performance level on cognitive performance in well-trained athletes. Evidence from previous studies indicates that endurance- and strength-training processes cause different adaptive changes at the CNS and peripheral levels (Leveritt et al., 1999). Taking this into account, one may argue that different types of exercise (endurance vs. strength) contribute to cognitive performance in a different way. At the CNS level, a plethora of changes in response to different types of exercise is particularly assigned to the lateral and dorsolateral prefrontal cortex, which takes part in regulating many human executive functions to prepare for situations demanding high levels of working memory, attention and cognitive flexibility (Arsten, 2011; Ludyga et al., 2016). This response can be attributed to the neural activation of dopaminergic, noradrenergic, glutaminergic systems and some neuropeptides (for example BDNF) which play a key role in activation of the prefrontal cortex and thereby cognitive function, as well as to periphery produced substances during exercise (for example lactate or ammonia) which can cross the blood-brain barrier (Arsten, 2011; Świątkiewicz et al., 2023; Zajac et al., 2015).

Our study reveals that exercise to volitional exhaustion (EVE) does not change CRT or NCR in strength-trained (S) athletes under both normoxic and hypoxic conditions. A similar phenomenon was also seen in our control (C) group. In turn, in endurance-trained (E) athletes, the EVE impaired CRT under normoxia and hypoxia (increases of 0.03 s and 0.05 s, respectively). Additionally, under hypoxia, the EVE reduced NCR by 1.7 compared to the resting state. Moreover, under hypoxia, NCR was lower in the E group after the EVE, as compared to the S (by 1.3) and C (by 1.2) groups.

On the basis of the values of maximal power reached by participants in the investigated groups, it can be assumed that exercise-induced changes in psychomotor performance occur only in response to heavily exhaustive exercise stress. This is evidenced in the present study by the rating of maximum relative (F = 3.517, *p* = 0.043) and absolute power that achieved the highest values in the E group as compared to the S and C groups (Table 1). In line with this assumption are the observed changes in catecholamine levels and some other biochemical and cardiovascular variables. Interestingly, the greatest increase in A and NA immediately after exercise was observed in the E group, under both hypoxia and normoxia. Moreover, under hypoxia, this increase was two and three times greater than under normoxia, respectively. At the same time, CRT was elongated immediately after maximal exercise under both normoxia and hypoxia; however, this change was significantly smaller under hypoxia compared to normoxia. In addition, the latter effect was accompanied by a reduction in NCR. Collectively, our results indicate that high-intensity exercise, performed during acute exposition to hypoxia, plays a dominant role in the pathomechanism of cognitive performance impairment. Previously, a similar effect was observed in healthy young people in response to interval exercise, as measured by the GNS task (Sun et al., 2019). Furthermore, the fact that only the E group showed cognitive impact of heavy exercise may reflect that these participants performed a higher overall motor activity (in the brain also) and exercised for a longer period of time than the other groups. The aforementioned influence on the brain could contribute to, and partially explain, differences between groups considering their cognitive function.

In our study, all groups were instructed to perform the maximal effort; therefore, we could expect similar effects at the CNS level. However, it has been shown that in endurance-trained athletes, a large percentage of exercise-induced fatigue originates from the so-called central fatigue (Tornero-Aquilera et al., 2022). In relation to our study, it can be assumed that E athletes terminated exercise more due to central fatigue than to peripheral fatigue, and this phenomenon was manifested by cognitive impairment. It is believed that strength-trained athletes are more sensitive to metabolic and chemical factors conducive to fatigue (Gandevia, 2001; Zajac et al., 2015). Hence, in our study S athletes could stop exercising without central fatigue, which resulted in no changes in cognitive function.

Consistent with the above reasoning are the catecholamines data in the E group. The higher workload in this group is associated with a significant activation of the sympathetic nervous system, both under H and N, which resulted in increased peripheral concentration of A and NA. Again, we can assume that the S and C groups stopped exercising before central fatigue occurred. Some data indicate that the effect of catecholamines on cognition depends on their level in the brain. Their release following moderate intensity exercise may promote cognitive function (Chmura et al., 1994), while an excess in A and NA can lead to the so-called neuronal noise, which is associated with inhibition of brain efficiency including cognitive impairment (McMorris et al., 2011, McMorris and Hale, 2012).

Animal studies have shown that stress induces A and DA signaling in the prefrontal cortex (Reader, 1981). Acute exposure to hypoxia has been found to be a stress factor in rats, leading to a decrease in cognitive performance, accompanied by an increase in A and DA (Miguel et al., 2018). Our study did not confirm that DA is an important player in this phenomenon, which is in line with previous research (Piotrowicz et al., 2020).

Excitatory synapses, most of all glutaminergic, dominate in the gray matter in the prefrontal cortex, indicating that excitatory neurotransmission gives rise to most of the energy requirements in this structure. Since evidence points to reduced cerebral blood flow under high intensity exercise, this can lead to reduced oxygen and glucose delivery from the vasculature into neurons and uncoupling between energy demand and supply. However, PET studies bring support to the notion that neuronal metabolic needs are met by glycolysis (Frigley and Stroman, 2011). Since the brain contains little energy reserves, the continued supply of energy substrates to neurons can be balanced by a large increase in LA production via the astrocyte-neuron lactate shuttle (Migistretti, 2009). This LA is released in the extracellular space (Bousier-Sore et al., 2006). What seems to be equally important, especially in our experimental setup, is the transport of LA from the blood via monocarboxylate transporters to the brain (Quistorff et al., 2008). In our study, the highest blood LA values in response to maximal exercise occurred in endurance-trained athletes both under hypoxic and normoxic conditions (11.6 mmol/l vs. 13.7 mmol/l, respectively), which created the most favorable conditions for the transport of this metabolite to the brain among the studied groups. A large body of evidence shows that neurons can efficiently use LA as an energy substrate (Boumezbeur et al., 2010), which is even preferentially metabolized over glucose when these substrates are simultaneously available (Itoh et al., 2003).

Interestingly, exercise-induced elevated BDNF levels did not prevent cognitive impairment in the E group under both normoxia and hypoxia conditions. Moreover, there was no effect on the improvement in CRT and NCR in the control group. Previous data indicated that one of the most important factors which can improve cognitive functions by exercise is the BDNF (Chang et al., 2012; Liu and Nusslock, 2018). However, they strongly relate to endurance training or prolonged aerobic exercises (Erickson et al., 2011; Huang and Reichard, 2009). It is believed that BDNF can improve cognition by binding to the TRKb receptor, triggering a neuroprotective cascade of intracellular signaling which involves the CaMK II, MAPK pathways (Małczyńska et al., 2019; McMorris and Hale, 2012). However, some authors suggest that the time to induce this positive effect is delayed (Slusher et al., 2018). Following this path, it would be expected that in our study, the improved CRT after rest under normoxia and hypoxia conditions was the result of the post- exercise BDNF increase. It should be noted that some authors did not report any relationship between BDNF changes and executive functions in relation to acute exercise (Piotrowicz et al., 2020; Slusher et al., 2018). Additionally, Slusher et al. (2018) showed improvement in cognitive functions after resting from short-interval exercise, but no correlation with the BDNF level, which could also confirm the delayed post-exercise effects of BDNF.

Another important finding is that the S group showed no increase in the BDNF as a result of the EVE under either normoxia or hypoxia. Under normoxia, the suppression of BDNF secretion could be explained by a significant increase in COR in this group. It is believed that stress is a factor releasing this hormone into the bloodstream (Choi et al., 2018), and its exercise level may be negatively correlated with the level of BDNF (Whiteman et al., 2014). Studies on animal models have shown that an increase in cortisol production caused a decrease in the production of BDNF in the brain (Dessypris et al., 1980; Zajac et al., 2010). It has been shown that the negative correlation can be also caused by a mutation in the BDNF encoding gene, resulting in a methionine (Met) occurrence in protein 66th residue (Val66Met) (Garcia-Suarez et al., 2020). Additionally, individuals with the aforementioned polymorphism may not show a response to exercise in the form of an increase in BDNF (Egan et al., 2003).

In the brain, ET-1 is produced by epithelial as well as neuronal and glial cells (among others), and is considered as the most potent vasoconstrictor and mitogen yet found (Silpanisong et al., 2017). ET-1 plays an essential role in the control of the brain microcirculation (Petrov et al., 2002). ET-1 stimulates secretion of nitric oxide and some other peptides participating in the hormonal control of salt and water balance (Levin, 1996). Under both physiological conditions and during exercise, ET-1 and NO play a pivotal antagonistic role in modulating the diameter of blood vessels (De Mey and Vanhutte, 2014; Rapoport and Merkus, 2018). Hypoxia and ischemia augment peripheral ET-1 secretion; however, their impact on brain ET-1 production and secretion is still poorly recognized (Levin, 1996). Importantly, during exercise, ET-1 is released to regulate vascular tone in areas that are not involved in exercise, thereby redirecting blood to organs involved in exercise, including the brain. In turn, increased exercise intensity may also lead to a balance between NO and ET-1 production due to pulsatile shear stress (De Mey and Vanhutte, 2014). The second factor enhancing production of the mentioned substances is hypoxia (Ogh et al., 2014). In our study, a significant increase in peripheral NO in the E and C groups during exercises under hypoxic condition may suggest an additive effect of these two factors in enhanced low body oxygen availability. This phenomenon is accompanied by an increase in the BDNF. The lack of significant changes in the level of ET-1 in our study indicates the lack of a meaningful role of this peptide in the regulation of cerebral microcirculation under hypoxia. This is in line with recent research indicating that acute intermittent hypoxia (IH) causes an increase in ET-1, muscle sympathetic nerve activity (MSNA) and blood pressure (BP) in healthy young men; however, the effects of IH on MSNA and BP do not occur via activation of ET receptors in untrained healthy young men (Limberg et al., 2022). The experimental evidence is more indicative of the conclusion that activated astrocytes are the most potent brain microcirculation regulators via termination ends located on significant portions of the intracerebral circulation (Nedergaard et al., 2002) and this cell population is well positioned to integrate neuronal activity and link neuronal activity to the vascular network (Ransom et al., 2003).

The correlation of BDNF production due to the NO signalization (Banoujaafar et al., 2016) is supported by the fact that in the C group, we did not observe an increase in either NO or BDNF due to the EVE. However, conversely, under normoxia, the exercise production of the aforementioned neurotrophin was increased despite there being no differences in NO concentration, which suggests that the BDNF can be synthesized independently of NO. Due to the fact that the increase in NO concentration in our study was not associated with the improvement in cognitive function in any group, it seems that this substance is not a major player in this phenomenon. It is worth mentioning that many studies which confirm the positive effect of NO on cognitive function were performed in vitro or after exogenic administration of NO (Lefferts et al., 2016). Moreover, Canossa et al. (2002) reported that only exogenously administered NO had a positive effect on the BDNF level and endogenous administration could suppress the production of this neurotrophin (Canossa et al., 2002).

## Conclusions

Maximal endurance exercise to volitional exhaustion impairs cognitive function only in endurance-trained young men, which is probably caused by the coexistence of peripheral and central fatigue. Higher involvement of the sympathetic nervous system expressed by elevated blood levels of A and NA in such athletes (compared to strength-trained athletes and individuals leading a sedentary lifestyle) may also play a role in this phenomenon. Additionally, exposure to moderate hypoxia did not have a negative impact on CRT or NCR. BDNF, which is known as the main factor enhancing cognitive function, in our study did not play an important role during exercise. Moreover, both NO and ET-1 seemed to have no effect on cognition, independent of oxygen availability (normoxia vs. moderate hypoxia) and the performance level.

## References

[ref1] Arsten, A. F. (2011). Catecholamine influences on dorsolateral prefrontal cortical networks. Biological Psychiatry, 69(12), e89–e99. 10.1016/j.biopsych.2011.01.027 21489408 PMC3145207

[ref2] Binder, D. K., & Scharfman, H. E. (2004). Brain-derived neurotrophic factor. Growth Factors, 22, 123–131. 10.1080/08977190410001723308 15518235 PMC2504526

[ref3] Boumezbeur, F., Petersen, K. F., Cline, G. W., Mason, G. F., Behar, K. L., Shulman, G. I., & Rothman, D. L. (2010). The contribution of blood lactate to brain energy metabolism in humans measured by dynamic 13C nuclear magnetic resonance spectroscopy. The Journal of Neuroscience, 30(42), 13983–13991. 10.1523/JNEUROSCI.2040-10.2010 20962220 PMC2996729

[ref4] Canossa, M., Giordano, E., Cappello, S., Guarnieri, C., & Ferri, S. (2002). Nitric oxide down regulates brain-derived neurotrophic factor secretion in cultured hippocampal neurons. Proceedings of the National Academy of Sciences of the United States of America, 99, 3282–3287. 10.1073/pnas.042504299 11867712 PMC122510

[ref5] Chang, Y. K., Labban, J. D., Gapin, J. I., & Etnier, J. L. (2012). The effects of acute exercise on cognitive performance: A meta-analysis. Brain Research, 1453, 87–101. 10.1016/j.brainres.2012.02.068 22480735

[ref6] Chmura, J., Nazar, K., & Kaciuba-Uscilko, H. (1994). Choice reaction time during graded exercise in relation to blood lactate and plasma catecholamine thresholds. International Journal of Sports Medicine, 15, 172–176. 10.1055/s-2007-1021042 8063464

[ref7] Choi, K. W., Na, E. J., Fava, M., Mischoulon, D., Cho, H., & Jeon H. J. (2018). Increased adrenocorticotropic hormone (ACTH) levels predict severity of depression after six months of follow-up in outpatients with major depressive disorder. Psychiatry Research, 270, 246–252. 10.1016/j.psychres.2018.09.047 30269042

[ref8] De Mey, J. G., & Vanhoutte, P. M. (2014). End O’ the line revisited: moving on from nitric oxide to CGRP. Life Sciences, 118, 120–128. 10.1016/j.lfs.2014.04.012 24747136

[ref9] Dessypris, A., Wägar, G., Fyhrquist, F., Mäkinen, T., Welin, M. G., & Lamberg, B. A. (1980). Marathon run: Effects on blood cortisol-ACTH, iodothyronines-TSH and vasopressin. Acta Endocrinologica (Copenh.), 95, 151–157. 10.1530/acta.0.0950151 6254304

[ref10] Dinoff, A., Herrmann, N., Swardfager, W., Liu, C.S., Sherman, C., Chan, S., & Lanctôt, K. L. (2016). The Effect of Exercise Training on Resting Concentrations of Peripheral Brain-Derived Neurotrophic Factor (BDNF): A Meta-Analysis. PLoS One, 11(9), e0163037. 10.1371/journal.pone.0163037 27658238 PMC5033477

[ref11] Egan, M. F., Kojima, M., Callicott, J. H., Goldberg, T. E., Kolachana, B. S., Bertolino, A., & Weinberger, D.R. (2003). The BDNF val66met polymorphism affects activity-dependent secretion of BDNF and human memory and hippocampal function. Cell, 112(2), 257–269. 10.1016/S0092-8674(03)00035-7 12553913

[ref12] Erickson, K. I., Voss, M. W., Prakas, R. S., Basak, C., Szabo, A., Chaddock, L., Kim, J. S., Heo, S., Alves, H., & White, S. M. (2011). Exercise Training Increases Size of Hippocampus and Improves Memory. Proceedings of the National Academy of Sciences of the United States of America, 108, 3017–3322. 10.1073/pnas.1015950108 21282661 PMC3041121

[ref13] Ferris, L. T., Williams, J. S., & Shen, C. L. (2007). The Efect of Acute Exercise on Serum Brain-Derived Neurotrophic Factor Levels and Cognitive Function. Medicine & Science in Sports & Exercise, 39(4), 728–734. 10.1249/mss.0b013e31802f04c7 17414812

[ref14] Frigley, C. R., & Stroman, P. W. (2011). The role(s) of astrocytes and astrocyte activity in neurometabolism, neurovascular coupling, and the production of functional neuroimaging signals. European Journal of Neuroscience, 33, 577-588. 10.1111/j.1460-9568.2010.07584.x 21314846

[ref15] Gandevia, S. C. (2001). Spinal and supraspinal factors in human muscle fatigue. Physiological Reviews, 81, 1725–1789. 10.1152/physrev.2001.81.4.1725 11581501

[ref16] Gibson, G. E., Duffy, & T. E. (1981). Impaired synthesis of acetylcholine by mild hypoxic hypoxia or nitrous oxide. Journal of Neurochemistry, 36, 28–33. 10.1111/j.1471-4159.1981.tb02373 7463052

[ref17] Gold, S. M., Schulz, K. H., Hartmann, S., Mladek, M., Lang, U. E., Hellweg, R., Reer, R., Braumann, K.M., & Heesen, C. (2003). Basal serum levels and reactivity of nerve growth factor and brain-derived neurotrophic factor to standardized acute exercise in multiple sclerosis and controls. Journal of Neuroimmunology, 138(1-2), 99–105. 10.1016/S0165-5728(03)00121-8 12742659

[ref18] Hashimoto, T., Tsukamoto, H., Ando, S., & Ogoh, S. (2021). Effect of Exercise on Brain Health: The Potential Role of Lactateas a Myokine. Metabolites, 11(12), 813. 10.3390/metabo11120813 34940571 PMC8709217

[ref19] Helm, E. E., Matt, K. S., Kirschner, K. F., Pohlig, R. T., Kohl, D., & Reisman, D.S. (2017). The influence of high intensity exercise and the Val66Met polymorphism on circulating BDNF and locomotor learning. Neurobiology of Learning and Memory, 144, 77–85. 10.1016/j.nlm.2017.06.003 28668279 PMC5583008

[ref20] Huang, E. J., & Reichardt, L. E. (2009). Neurotrophins: Roles in neuronal development and function. Annual Review of Neuroscience, 24, 677–736. 10.1146/annurev.neuro.24.1.677 PMC2758233 11520916

[ref21] Hung, C. L., Tseng, J. W., Chao, H. H., Hung, T. M., & Wang, H. S. (2018). Effect of acute exercise mode on serum brain-derived neurotrophic factor (BDNF) and task switching performance. Journal of Clinical Medicine, 7(10), 301. 10.3390/jcm7100301 30249981 PMC6209934

[ref22] Itoh, Y., Esaki, T., Shimoji, K., Cook, M., Law, M. J., Kaufman, E., & Sokoloff, L. (2003). Dichloroacetate effects on glucose and lactate oxidation by neurons and astroglia in vitro and on glucose utilization by brain in vivo. Proceedings of the National Academy of Sciences of the United States of America, 100, 4879–4884. 10.1073/pnas.0831078100 12668764 PMC153649

[ref23] Jiménez-Maldonado, A., Rentería, I., García-Suárez, P. C., Moncada-Jiménez, J., & Freire-Royes, L. F. (2018). The impact of high-intensity interval training on brain derived neurotrophic factor in brain: A mini-review. Frontiers in Neuroscience, 12, 839. 10.3389/fnins.2018.00839 30487731 PMC6246624

[ref24] Kolb, J. C., Ainslie, P. N., Ide. K., & Poulin, M. J. (2004). Protocol to measure acute cerebrovascular and ventilatory responses to isocapnic hypoxia in humans. Respiratory Physiology & Neurobiology, 141(2), 191–199. 10.1016/j.resp.2004.04.014 15239969

[ref25] Komiyama, T., Katayama, K., Sudo M., Ishida, K., Higaki, Y., & Ando, S. (2017). Cognitive function during exercise under severe hypoxia. Scientific Reports, 7, 10000. 10.1038/s41598-017-10332-y 28855602 PMC5577198

[ref26] Kramer, A. F., Erickson, K. I., & Colcombe, S. J. (2006). Exercise, cognition, and the aging brain. Journal of Applied Physiology, 101(4), 1237–42. 10.1152/japplphysiol.00500.2006 16778001

[ref27] Kuipers, H., Verstappen, F. T. J., Keizer, H. A., Guerten, P., & van Kranenburg, G. (1985). Variability of aerobic performance in the laboratory and its physiological correlates. International Journal of Sports Medicine, 6, 197–201. 10.1055/s-2008-1025839 4044103

[ref28] Langfort, J., Barańczuk, E., Pawlak, D., Chalimoniuk, M., Lukacova, N., Marsala, J., & Górski, J. (2006). The effect of endurance training on regional serotonin metabolism in the brain during early stage of detraining period in the female rat. Cellular and Molecular Neurobiology, 26(7-8), 1327–42. 10.1007/s10571-006-9065-5 16897368 PMC11520764

[ref29] Lefferts, W. K., Hughes, W. E., White, C. N., Brutsaert, T. D., & Heffernan, K. S. (2016). Effect of acute nitrate supplementation on neurovascular coupling and cognitive performance in hypoxia. Applied Physiology, Nutrition, and Metabolism, 41, 133–141. 10.1139/apnm-2015-0400 26751937

[ref30] Leveritt, M., Abernethy, P. J., Barry, B. K., & Logan, P. A. (1999). Concurrent strength and endurance training. A review. Sports Medicine, 28(6), 413–27. 10.2165/00007256-199928060-00004 10623984

[ref31] Levin, E. R. (1996). Endothelins as cardiovascular peptides. American Journal of Nephrology, 16(3), 246–51. 10.1159/000169004 8739884

[ref32] Levitt, S., & Gutin, B. (1971). Multiple-choice reaction time and movement time during physical exertion. Research Quarterly for Exercise and Sport, 42, 406–410. 5291431

[ref33] Limberg, J. K., Baker, S. E., Ott, E. P., Jacob, D. W., Scruggs, Z. M., Harper, J. L., & Manrique-Acevedo, C.M. (2022). Endothelin-1 receptor blockade does not alter the sympathetic and hemodynamic response to acute intermittent hypoxia in men. Journal of Applied Physiology (1985), 133(4), 867-875. 10.1152/japplphysiol.00837.2021 PMC9560055 35952348

[ref34] Liu, P. Z., & Nusslock, R. (2018). Exercise-Mediated Neurogenesis in the Hippocampus via BDNF. Frontiers in Neuroscience, 12, 52. 10.3389/fnins.2018.00052 29467613 PMC5808288

[ref35] Ludyga, S., Gerber, M., Brand, S., Holsboer-Trachsler, E., & Puhse, U. (2016). Acute Effects of Moderate Aerobic Exercise on Specific Aspects of Executive Function in Different Age and Fitness Groups: A Meta-Analysis. Psychophysiology, 53, 1611–1626. 10.1111/psyp.12736 27556572

[ref36] Małczyńska, P., Piotrowicz, Z., Drabarek, D., Langfort, J., & Chalimoniuk, M. (2019). The role of the brain-derived neurotrophic factor (BDNF) in neurodegenerative processes and in the neuroregeneration mechanisms induced by increased physical activity. Postępy Biochemii, 65, 2–8. 10.18388/pb.2019_251 30901514

[ref37] McMorris, T., & Hale, B. J. (2012). Differential effects of differing intensities of acute exercise on speed and accuracy of cognition: A meta-analytical investigation. Brain and Cognition, 80(3), 338–51. 10.1016/j.bandc.2012.09.001 23064033

[ref38] McMorris, T., Sproule, J., Turner, A., & Hale, B. J. (2011). Acute, intermediate intensity exercise, and speed and accuracy in working memory tasks: A meta-analytical comparison of effects. Physiology and Behavior, 102, 421–428. 10.1016/j.physbeh.2010.12.007 21163278

[ref39] Migistretti, P. J. Role of glutamate in neuron-glia metabolic coupling. (2009). The American Journal of Clinical Nutrition, 90, 875S-880S. 10.3945/ajcn.2009.27462CC 19571222

[ref40] Miguel, P. M., Deniz, B. F., Deckmann, I., Confortim, H. D., Diaz, R., Laureano, D. P., Silveira, P. P., & Pereira, L. O. (2018). Prefrontal Cortex Dysfunction in Hypoxic-Ischaemic Encephalopathy Contributes to Executive Function Impairments in Rats: Potential Contribution for Attention-Deficit/ Hyperactivity Disorder. The World Journal of Biological Psychiatry, 19, 547–560. 10.1080/15622975.2016.1273551 28105895

[ref41] Nedergaard, M., Takano, T., & Hansen, A. J. (2002). Beyond the role of glutamate as a neurotransmitter. Nature Reviews Neuroscience, 3(9), 748–55. 10.1038/nrn916 12209123

[ref42] Ochi, G., Kanazawa, J., Hyodo, K., Suwabe, K., Shimizu, T., Fukuie, T., Byun, K., & Soya, H. (2018). Hypoxia-induced lowered executive function depends on arterial oxygen desaturation. The Journal of Physiological Sciences, 68, 847–853. 10.1007/s12576-018-0603-y 29536370 PMC10717617

[ref43] Ogh, S., Tsukamoto, H., Hirasawa, A., Hasegawa, H., Hirose, N., & Hashimoto T. (2014). The Effect of Changes in Cerebral Blood Flow on Cognitive Function during Exercise. Physiological Reports, 28, 2(9), e12163. 10.14814/phy2.12163 PMC4270220 25263210

[ref44] Petrov, T., Steiner, J., Baun, B., & Rafols, J. A. (2002). Sources of endothelin-1 in hippocampus and cortex following traumatic brain injury. Neuroscience, 115(1), 275–83. 10.1016/S0306-4522(02)00345-7 12401340

[ref45] Piotrowicz, Z., Chalimoniuk, M., Płoszczyca, K., Czuba, M., & Langfort J. (2020). Exercise-Induced Elevated BDNF Level Does Not Prevent Cognitive Impairment Due to Acute Exposure to Moderate Hypoxia in Well-Trained Athletes. International Journal of Molecular Sciences, 21(15), 5569. 10.3390/ijms21155569 32759658 PMC7432544

[ref46] Piotrowicz, Z., Chalimoniuk, M., Płoszczyca, K., Czuba, M., & Langfort, J. (2019). Acute normobaric hypoxia does not affect the simultaneous exercise-induced increase in circulating BDNF and GDNF in young healthy men: A feasibility study. PLoS One, 14(10), e0224207. 10.1371/journal.pone.0224207 31644554 PMC6808427

[ref47] Quistorff, B., Secher, N. H., & Van Lieshout, J. J. (2008). Lactate fuels the human brain during exercise. The FASEB Journal, 22(10), 3443–9. 10.1096/fj.08-106104 18653766

[ref48] Rapoport, R. M., & Merkus, D. (2018). Endothelin-1 Regulation of Exercise-Induced Changes in Flow: Dynamic Regulation of Vascular Tone. Frontiers in Pharmacology, 8, 517. 10.3389/fphar.2017.00517 PMC5660699 29114220

[ref49] Reader, T. A. (1981). Distribution of catecholamines and serotonin in the rat cerebral cortex: absolute levels and relative proportions. Journal of Neural Transmission, 50(1), 13–27. 10.1007/BF01254910 7205246

[ref50] Seo, Y., Gerhart, H. D., Stavres, J., Fennell C., Draper, S., & Glickman, E. L. (2017). Normobaric Hypoxia and Submaximal Exercise Effects on Running Memory and Mood State in Women. Aerospace Medicine and Human Performance, 88(7), 627–632. 10.3357/AMHP.4798.2017 28641679

[ref51] Silpanisong, I., Kim, D., Williams, J. M., Adeoye, O. O., Thorpe, R. B., & Pearce, W. J. (2017). Chronic hypoxia alters fetal cerebrovascular responses to endothelin-1. American Journal of Physiology Cell Physiology, 313(2), C207–C218. 10.1152/ajpcell.00241.2016 PMC5582873 28566491

[ref52] Slusher, A. L., Patterson, V. T., Schwartz, C. S., & Acevedo, E. O. (2018). Impact of high intensity exercise on execrative function and brain derived neurotrophic factor in healthy college aged males. Physiology & Behavior, 191, 116–122. 29673858 10.1016/j.physbeh.2018.04.018

[ref53] Sun, S., Loprinzi, P. D., Guan, H., Zou, L., Kong, Z., Hu, Y., Shi, Q., & Nie J. (2019). The Effects of High-Intensity Interval Exercise and Hypoxia on Cognition in Sedentary Young Adults. Medicina (Kaunas), 55(2), 43. 10.3390/medicina55020043 30744172 PMC6409841

[ref54] Świątkiewicz, M., Gaździński, S., Madeyski, M., Kossowski, B., Langfort, J., Bogorodzki, P., Zawadzka-Bartczak, E., Sklinda, K., Walecki, J., & Grieb, P. (2023). Increased brain 1H-MRS glutamate and lactate signals following maximal aerobic capacity exercise in young healthy males: an exploratory study. Biology of Sport, 40(3), 665–673. 10.5114/biolsport.2023.118335 37398967 PMC10286605

[ref55] Szuhany, K. L., Bugatti, M., & Otto, M. W. (2015). A meta-analytic review of the effects of exercise on brain-derived neurotrophic factor. Journal of Psychiatric Research, 60, 56–64. 10.1016/j.jpsychires.2014.10.003 25455510 PMC4314337

[ref56] Taylor, L., Watkins, S. L., Marshall, H., Dascombe, B. J., & Foster, J. (2016). The impact of different environmental conditions on cognitive function: a focused review. Frontiers in Physiology, 6, 372. 10.3389/fphys.2015.00372 26779029 PMC4701920

[ref57] Tornero-Aguilera, J. F., Jimenez-Morcillo, J., Rubio-Zarapuz, A., & Clemente-Suárez, V. J. (2022). Central and Peripheral Fatigue in Physical Exercise Explained: A Narrative Review. International Journal of Environmental Research and Public Health, 19(7), 3909. 10.3390/ijerph19073909 35409591 PMC8997532

[ref58] Urbaniak, G. C., & Plous, S. (2013). Research Randomizer (Version4.0) [Computer Software]. Available online: http://www.randomizer.org/ (accessed on 25 March 2017).

[ref59] Van Cutsem, J., Pattyn, N., Vissenaeken, D., Dhondt, G., De Pauw, K., Tonoli, C., Meeusen, R., & Roelands, B. (2015). The influence of a mild thermal challenge and severe hypoxia on exercise performance and serum BDNF. European Journal of Applied Physiology, 115, 2135–2148. 10.1007/s00421-015-3193-x 26026261

[ref60] Ventriglia, M., Zanardini, C. B., Zanetti, O., Volpe, D., Pasqualetti, P., Gennarelli, M., & Bocchio-Chiavetto, L. (2013) Serum brain-derived neurotrophic factor levels in different neurological diseases. BioMed Research International, ID 901082. 10.1155/2013/901082 24024214 PMC3760208

[ref61] Vilela, T. C., Muller, A. P., Damiani, A. P., Macan, T. P., da Silva, S., Canteiro, P. B., de Sena Casagrande, A., Pedroso, G. D. S., Nesi, R. T., de Andrade, V. M., & de Pinho, R. A. (2017). Strength and Aerobic Exercises Improve Spatial Memory in Aging Rats Through Stimulating Distinct. Molecular Neurobiology, 54, 7928–7937. 10.1007/s12035-016-0272-x 27878552

[ref62] Whiteman, A. S., Young, D. E., He, X., Chen, T. C., Wagenaar, R. C., Stern, C. E., & Schon, K. (2014). Interaction between serum BDNF and aerobic fitness predicts recognition memory in healthy young adults. Behavioural Brain Research, 259, 302–312. 10.1016/j.bbr.2013.11.023 24269495 PMC3991014

[ref63] Williams, J. S., & Ferris, L. T. (2012). Effects of endurance exercise training on brain-derived neurotrophic factor. Journal of Exercise Physiology Online, 15(4), 11–17.

[ref64] Wilson, M. H., Newman, S., & Imray, C. H. (2009). The cerebral effects of ascent to high altitudes. The Lancet Neurology, 8(2), 175-91. 10.1016/S1474-4422(09)70014-6 19161909

[ref65] Zajac, A., Chalimoniuk, M., Maszczyk, A., Gołaś, A., & Langfort, J. (2015). Central and Peripheral Fatigue During Resistance Exercise-A Critical Review. Journal of Human Kinetics, 49,159–69. 10.1515/hukin-2015-0118 26839616 PMC4723165

[ref66] Zajac, A., Poprzęcki, S., Zebrowska, A., Chalimoniuk, M., & Langfort, J. (2010). Arginine and ornithine supplementation increases growth hormone and insulin-like growth factor-1 serum levels after heavy-resistance exercise in strength-trained athletes. Journal of Strength and Conditioning Research, 24(4), 1082–1090. 10.1519/JSC.0b013e3181d321ff 20300016

[ref67] Zoladz, J. A., & Pilc, A. (2010). The effect of physical activity on the brain derived neurotrophic factor: from animal to human studies. Journal of Physiology and Pharmacology, 61, 533–541. 21081796

